# Genome-wide characterization of the NRAMP gene family in *Phaseolus vulgaris* provides insights into functional implications during common bean development

**DOI:** 10.1590/1678-4685-GMB-2017-0272

**Published:** 2018-10-11

**Authors:** Juliane Karine Ishida, Danielle G.G. Caldas, Lucas Roberto Oliveira, Gabriela Campos Frederici, Lucas Margato Pereira Leite, Tsai Siu Mui

**Affiliations:** ^1^ Centro de Energia Nuclear na Agricultura, Universidade de São Paulo (CENA-USP), Piracicaba, SP, Brazil

**Keywords:** Phaseolus vulgaris, transporter, NRAMP, gene family, phylogenetic analysis

## Abstract

Transporter proteins play an essential role in the uptake, trafficking and storage of metals in plant tissues. The Natural Resistance-Associated Macrophage Protein (NRAMP) family plays an essential role in divalent metal transport. We conducted bioinformatics approaches to identify seven *NRAMP* genes in the *Phaseolus vulgaris* genome, investigated their phylogenetic relation, and performed transmembrane domain and gene/protein structure analyses. We found that the *NRAMP* gene family forms two distinct groups. One group included the *PvNRAMP1*, *-6,* and *-7* genes that share a fragmented structure with a numerous exon/intron organization and encode proteins with mitochondrial or plastidial localization. The other group is characterized by few exons that encode cytoplasmic proteins. In addition, our data indicated that *PvNRAMP6 and -7* may be involved in mineral uptake and mobilization in nodule tissues, while the genes *PvNRAMP1, -2, -3, -4* and *-5* are potentially recruited during plant development. This data provided a more comprehensive understanding of the role of NRAMP transporters in metal homeostasis in *P. vulgaris*.

## Introduction

The common bean (*Phaseolus vulgaris* L.) is one of the most consumed crops worldwide (www.fao.org/faostat). It is rich in protein, carbohydrates, and minerals (Gepts *et al.*, 2008) and its frequent consumption may prevent illnesses like cardiovascular diseases (Bouchenak and Lamri-Senhadji, 2013), colon cancer (Bennink, 2002), and diabetes (Geil and Anderson, 1994). In the developing world, bean is a main source of nutrition. For example, in sub-Saharan Africa, one of the most critical areas for food security, beans represent 15% of the calories and 36% of the proteins in dairy supplies (www.fao.org/faostat). In Latin America, the per capita beans consumption is around 16 kg/year (Broughton *et al.*, 2003). The nutritional benefits of consuming beans require a deeper understanding of the molecular mechanisms involved in nutrient accumulation and mobilization in plant tissues, especially in the seeds.

The identification of a specific set of transporters that provides the fine balance of metal concentration across the cellular membrane is essential to understanding the physiological function of these elements in plant development (Dordas, 2008). One relevant transporter family is NRAMP (Natural Resistance-Associated Macrophage Protein). NRAMP genes encode integral membrane proteins responsible for the transport of divalent metals across the cellular membrane. The NRAMP family is highly conserved from bacteria to mammals, showing a preserved consensus motif and 10–12 transmembrane domains (Cellier *et al.*, 1995). The emergence of NRAMPs occurs parallel to the increase in atmospheric oxygen content (Cellier *et al.*, 2001), and it may have evolved from the LeuT superfamily cation-driven transporter (Cellier 2012) in a putative anaerobic prokaryote ancestor, probably close to the *Chlorobium* group (Cellier *et al.*, 2001). In prokaryotes, the biological function of NRAMP is associated with defense and nutrition (Nevo and Nelson, 2006), while in animals the primary function of NRAMP lies in the maintenance of iron homeostasis (Gunshin *et al.*, 2001; Iolascon and De Falco, 2009; Shawki *et al.*, 2012; Victoria *et al.*, 2012). The plant NRAMP family is responsible for keeping the right balance of divalent ions (Fe^2+^, Mn^2+^, Cu^2+^, and Zn^2+^) (Sebastien and Schroeder, 2004; Victoria *et al.*, 2012) and the trivalent (Al^3+^) ion (Xia *et al.*, 2010) across the plasma and vascular membranes. Phylogenetic data revealed the existence of two NRAMP subgroups (Sebastien and Schroeder 2004): one obtained from a bacterial genome during the endosymbiotic process that originated double-membrane organelles; and another group that shares high amino acid identity/similarity with proteins found in the animal genome, raising the hypothesis of multiple origins of the NRAMP family (Sebastien and Schroeder, 2004).

There are six NRAMP genes in *Arabidopsis* (Thomine *et al.*, 2000). Mutants missing the vacuole membrane proteins AtNRAMP3 and -4 show a reduction in germination rate under iron deficient conditions (Lanquar *et al.*, 2005) and an impairment in photosynthesis capacity if manganese is limited (Lanquar *et al.*, 2010). Additionally, AtNRAMP1 is a key transporter for iron homeostasis (Curie *et al.*, 2000). Rice contains seven members of the NRAMP family (Belouchi *et al.*, 1997). OsNRAMP1 translocates the beneficial Fe^2+^ and also the toxic arsenic (As^3+^) and cadmium (Cd^2+^) in rice (Takahashi *et al.*, 2011; Tiwari *et al.*, 2014). The transporters OsNRAMP4 and OsNRAMP5 play a role in the intracellular mobilization of Al^3+^ (Xia *et al.*, 2010) and Mn^2+^/ Cd^2+^ (Ishimaru *et al.*, 2012), whereas OsNRAMP3 is present in conducting vascular cells and responsible for allocating Mn^2+^ from source to sink tissues. Apparently, OsNRAMP3 does not act in Fe^2+^ translocation (Yamaji *et al.*, 2013). Similarly, the HvNRAMP5 in barley enables the uptake of Mn^2+^ and the toxic Cd^2+^, but not of Fe^2+^(Wu *et al.*, 2016). In the hyper accumulating species *Thlaspi caerulescens* and *Thlaspi japonicum,* the NRAMP protein is responsible for mobilizing the heavy metals Cd^2+^ and Ni^2+^ in plant tissues (Mizuno *et al.*, 2005; Oomen *et al.*, 2009).

A synapomorphic trait of Fabaceae is the symbiotic association with nitrogen-fixing bacteria at specialized root organs, the nodules (Gage, 2004). There are two types of nodules in leguminous plants with different developmental strategies. The determinate type found in *P. vulgaris* and soybean *(Glycine max)* roots is characterized by a spherical shape due to the reduction of activity in meristematic tissues shortly after nodule initiation (Gage, 2004). The indeterminate nodules present in roots of *Medicago truncatula* and peas have a typically elongated shape. The meristematic region is continuously producing new cells along the active period of the nodule, generating different zones related to the developmental stage of symbiotic bacterial cells (bacterioid) (Jones *et al.*, 2007). In Zone I, the meristem actively forms new nodule tissues. In Zones II and III occur the infection and nitrogen fixation, and in Zone IV (closer to the root) the bacteroid starts to degenerate (Jones *et al.*, 2007). A better comprehension of nodule formation may provide insights towards employing bacteria to increase the availability of nitrogen in the soil, hence reducing the need for fertilizers. Iron is a required micronutrient for establishing symbiosis with nitrogen-fixing bacteria, since it is a cofactor of the enzyme nitrogenase recruited for atmospheric nitrogen uptake (Brear *et al.*, 2013). In *M. truncatula*, the protein MtNRAMP1 presented high expression in the nodule region, acting as an iron transporter across the plasma membrane during nodulation (Tejada-Jiménez *et al.*, 2015). In *G. max*, a NRAMP member (GmDMT1) located in the symbiosome membrane is responsible for the iron absorption in rhizobia-symbiotic tissues (Kaiser *et al.*, 2003). Thus, the NRAMP transporters are apparently of relevance in the import of nutrients for nodule formation, regardless of whether they are of a determinate or indeterminate type.

The availability of a fully sequenced genome for common bean provides opportunities to characterize gene transporter families and to identify their potential roles in uptake, allocation and storage of soil minerals in plant tissue. A better understanding of dynamics metal ions is an important step to improve crop breeding. So far, information on the major proteins involved in the transport of metals in common bean remains incipient. Here we identified and characterized seven homologs of NRAMP transporters in *P. vulgaris*. The gene structure, chromosomal distribution, phylogenetic tree, and the expression of NRAMP homologs were investigated using bioinformatics approaches. The results obtained contribute to comprehend the biological role of the players involved in metal homeostasis during *P. vulgaris* development.

## Material and Methods

### Identification and annotation of NRAMP genes in *P. vulgaris* genome

The keyword NRAMP was used to search the Phytozome database (http://www.phytozome.net) in the *Arabidopsis thaliana* genome (TAIR10). AtNRAMP sequences were identified and used as query to identify their protein homologs in the *P. vulgaris* genome (G19833). All sequences with a similarity score above 50% were considered. To search for *P. vulgaris* NRAMP genes we used the web tool TMHMM server (http://cbs.dtu.dk)(Krogh *et al.*, 2001) to predict transmembrane domains in putative protein sequences and the TargetP 1.1 Server (Emanuelsson *et al.*, 2007) for the prediction of subcellular localization. The general information of NRAMP genes, including the genomic, cDNA, and coding sequences, protein length, etc., were extracted from the Phytozome website (www.phytozome.jgi.doe.g.,ov). The exon/intron organization was accessed using the online tool GSDS 2.0 (http://gsds.cbi.pku.edu.cn) (Hu *et al.*, 2015), and the similarities among exons were compared using the BLASTN algorithm (Altschul 1997). The Ka/Ks ratio was calculated using the option Compute Pairwise Distances in MEGA7 software (Kumar *et al.*, 2016); the analysis was conducted using the Nei-Gojobori Method with all the positions containing gaps and missing values eliminated.

### Chromosome distribution, syntenic and regulatory elements analysis of common bean NRAMP genes

The chromosomal localization and putative promoter regions (3 kb from ATG site) of *PvNRAMP* genes were extracted using the software GENEIOUS® (www.geneious.com). All figures were edited using the Adobe Illustrator package. The *cis*-elements of promoter sequences and IRE motifs were identified by PlantCARE (Lescot *et al.*, 2002) and SIRE programs (Campillos *et al.*, 2010), respectively, under default parameters.

### Model structure and phylogenetic tree construction of NRAMPs

The putative protein sequences were aligned using the CLUSTALW (Thompson *et al.*, 2002) algorithm with default settings, and the phylogenetic trees were generated using MEGA7 software (Kumar *et al.*, 2016) with the Maximum Likelihood statistical method set to a 10,000 bootstrap value. Protein structures of NRAMPs were predicted using the Swiss-model software based on data of the *Staphylococcus capitis* divalent ion transporter (DMT) complex with manganese (ID: 4wgw.1). Putative models were visualized and edited using the Ras Top Molecular Visualization Software (www.geneinfinity.org/rastop).

### RNA-Seq analysis of NRAMP genes

The gene expression atlas data was obtained from the website www.plantgrn.noble.org/PvGEA. The expression levels were obtained from the Phytozome website, and RNA-Seq data of *P. vulgaris* embryo were retrieved from public database under the code GSE57535. To download the large dataset in Phytozome a login and password were created for access to the JG1 genome portal, and the latest version of the *P. vulgaris* transcriptome was downloaded for analysis (*P. vulgaris*_442_ver2.1). Heatmaps were generated using the GenePattern 2.0 program (Reich *et al.*, 2006) with a hierarchical algorithm. Distance measures for column and raw clustering were performed using the Pearson’s correlation coefficient (absolute value) with the Pairwise Average-Linkage method, which calculates the distance between two clusters based on the average distance between elements located in the two clusters. Expression levels of NRAMP genes in *M. truncatula* were obtained from PLEXdb (www.plexdb.org), a database for gene expression resources for plants and pathogens.

### *P. vulgaris* infection by *Colletotrichum lindemuthianum*.

*Colletotrichum lindemuthianum* (race 73) was cultivated in MATHUR medium (8 g/L dextrose, 2.5 g/L MgSO_4_.7H_2_O, 2.7 g/L KH_2_PO_4_, 2.4 g/L peptone, 2 g/L yeast extract, and 16 g/L agar) at 28 °C in darkness until sporulation. The medium surface was scraped with the aid of a Drigalsky loop and the spores suspended in 40 mL of sterile Milli-Q water containing 0.01% (v/v) Tween 20. The spore concentration was adjusted to 1.0 x 10^6^ cells/mL using a Neubauer chamber. *P. vulgaris* seeds (genotype SEL1308) were immersed for 10 min in 10% (v/v) sodium hypochlorite solution, followed by five washes with sterile water. The seeds were germinated on a wet filter paper in darkness. Plants were grown in a vermiculate/sand (1:1) substrate at 22 °C under a photoperiod of 16 h light and 8 h dark. First trifoliate leaves from 10-day-old seedlings were inoculated with a drop of the *C. lindemuthianum* spore suspension (1.0 x 10^6^ cells/mL). Simultaneously, the same number of plants was inoculated with a solution of 0.01% Tween 20 diluted in sterile water. Plants were covered with colorless and translucent plastic bags to keep the humidity close to 100%. Inoculated-leaf pieces (5 x 5 mm) were collected 65 hours after the fungal infection (65 hai), using a razor blade. They were immediately frozen in liquid nitrogen and stored at -80 °C. The experiment was conducted with biological triplicates.

### RNA extraction and qRT-PCR analysis

Total RNA from all samples was extracted using a NucleoSpin RNA XS kit (Macherey-Nagel). First strand cDNAs were synthesized from 0.1 μg of total RNA following the instructions for the MAXIMA First Strand cDNA synthesis kit (Fermentas). As internal control for the qRT-PCR assays we used the primer pairs IDE and Act11 (Borges *et al.*, 2012) which are specific for transcripts encoding an insulin degrading enzyme and actin, respectively. The primers for *PvNRAMP* genes were designed using Primer3Plus software (Untergasser *et al.*, 2007) under default qPCR settings, as listed in Table S1. The qRT-PCR assays were carried out in a StepOnePlus Real Time PCR System (Applied Biosystems) using 5 μL of SYBR® Green 2X Mix (Thermo-Fisher), 250 nM of each primer and 100 ng of cDNA, in a total volume of 10 μL. The amplification condition was 95 °C for 10 min, 40 cycles of 95 °C for 15 s, 59 °C for 20 s and 72 °C for 30 s, A final step of 15 s at 95°C, 1 min at 59°C and fluorescence measure at each 0.7°C variation (from 60 °C to 95 °C) was included to obtain the melting curve. Two to three technical replicates were performed.

To calculate the *PvNRAMP* relative expression, the raw data of fluorescence levels were submitted to LinRegPCR software (Ramakers *et al.*, 2003) to establish the baseline correction and run a linear regression analysis on each amplification curve. Next, the optimal set of data points (Window-of-Linearity) was defined for each amplification data set to allow the calculation of the threshold and quantification cycle (Cq) values for each sample. The reaction efficiency was calculated based on slope of the line (E = 10^slope^), considering an ideal value range (1.8 ≤ E ≤ 2) and a correlation of R ≥ 0.995. Expression comparisons were done using the Pair Wise Fixed Reallocation Randomization test (with bootstrap = 2000 permutations), implemented in the Relative Expression Software Tool (REST^©^- 384 version 2) (http://www.gene-quantification.info) as described by (Pfaffl *et al.*, 2002).

## Results

### Identification and classification of *NRAMP* genes in the *P. vulgaris* genome

**Figure 1:**
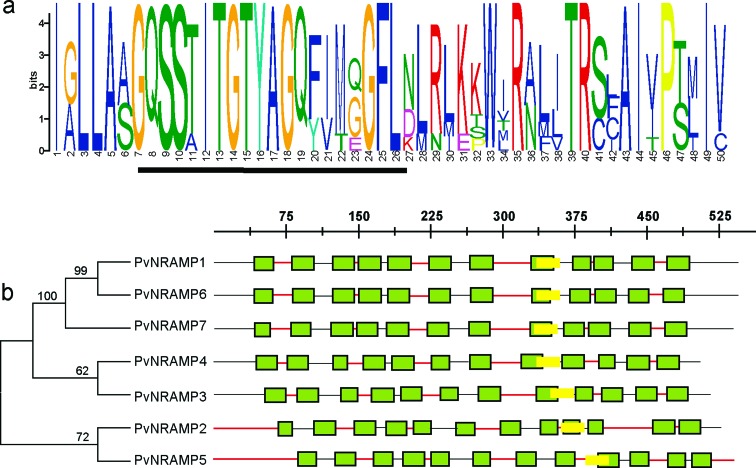
Diagrammatic representation of architecture of the NRAMP putative protein in *P. vulgaris*. (a) The conserved motif graph generated by the MEME program, displayed by stacks of letters at each site. The conserved amino acid is marked by a yellow box at the bottom of stacked letters. (b) Schematic view of transmembrane domain (TM) (green), the extracellular and intracellular portions are represented in black and red lines, respectively. Conserved motif between TM 8 and 9 is shown in yellow. The scale is set to the number of amino acids.

Nine sequences were initially found in the *P. vulgaris* genome using as query the homologs of *A. thaliana* NRAMP genes. Among these, only seven genes carried the consensus residues GQSSTITGTYAGQFIMGGFLN (Figure 1a), a unique motif associated with the NRAMP metal transporter family (Cellier *et al.*, 1995). To facilitate understanding we renamed the NRAMP homologs in *P. vulgaris* as *PvNRAMP1 to -7*. The corresponding Phytozome code, gene length, and protein length are summarized in Table 1. They encode putative proteins ranging from 507 to 554 amino acid residues in length, with an average of 58.5 kDa of molecular mass and a deduced isoelectric point (pI) ranging from 4.81 to 7.89 (Table 1). The genes *PvNRAMP1*, *-6,* and *-7* encode proteins with a basic pI and with plastid localization, while the genes *PvNRAMP2*, *-3*, *-4,* and *-5* generate a cytoplasmic protein with acid pI (Table 1). Twelve transmembrane domains (TM) were verified for all the members, with N- and C- tails pointing to opposite sides of the plasma membrane for *PvNRAMP2*, and C- and N-terminal turned to the intracellular region for *PvNRAMP5* and the extracellular side for other *PvNRAMPs* (Figure 1b). For all the *PvNRAMPs*, the conservative motif was localized between TM 8 and 9 (Curie *et al.*, 2000; Hall and Williams, 2003), placed between 401–418 and 431–453 aa, similar to previously characterized NRAMP genes in plants (Kaiser *et al.*, 2003; Mizuno *et al.*, 2005; Vatansever *et al.*, 2016).

**Table 1:** *P. vulgaris NRAMP* gene family overall features.

Name	Phytozome ID	Genomic length (bp)	Transcript length (bp)	CDS length (bp)	Chr No.	Putative protein (aa)	kDa	PI	Subcellular localization prediction
PvNRAMP1	Phvul.005G182000	6104	2089	1635	5	544	59	7.4	Chloroplast
PvNRAMP6	Phvul.010G160800	5423	1933	1635	10	544	59.1	7.1	Chloroplast
PvNRAMP7	Phvul.009G127900	4370	2010	1617	9	538	58	7.9	Mitochondrion
PvNRAMP2	Phvul.009G069700	4071	2456	1587	9	528	57.8	5.5	-
PvNRAMP3	Phvul.003G238600	3960	2026	1551	3	516	56.7	5.2	-
PvNRAMP4	Phvul.002G014300	3638	2210	1524	2	507	55.7	5.1	-
PvNRAMP5	Phvul.010G110500	3697	1665	1665	10	554	61.1	4.8	-

### Gene structure and chromosomal distribution of *NRAMP* gene in *P. vulgaris*

**Figure 2:**
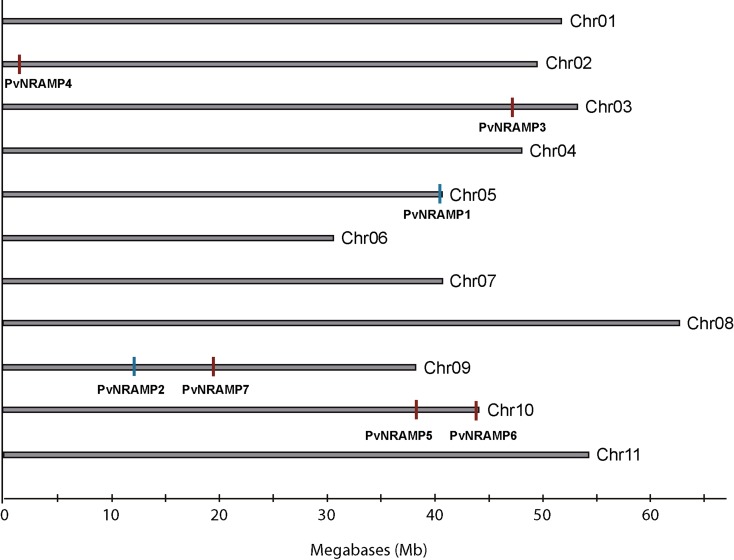
Distribution of genes in *P. vulgaris* chromosome. Chromosome size is indicated by its relative length. The scale on the bottom is shown in megabases (Mb). The blue bars mark the gene location at the forward direction, while the red bars mark the gene at the reverse position. The figure was generated by Geneious software and edited using the package Adobe Illustrator.

**Figure 3:**
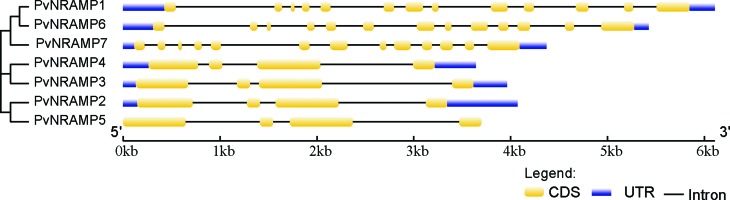
Schematic structure of *PvNRAMP* in *P. vulgaris*. Phylogenetic tree with introns, exons and UTRs. Lines connected to similar exons and different colors indicate novel exons. The phylogenetic tree was generated based on the Maximum Likelihood statistical method and the phylogenetic tree must show the numbers at the internal nodes.

To investigate the evolutionary relationship among *NRAMP* genes in *P. vulgaris*, their chromosome map was constructed (Figure 2). The *PvNRAMP1*, *-3* and *-4* genes were localized in chromosome 5, 3, and 2, respectively. The genes *PvNRAMP2* and *-7* were at chromosome 9, while *PvNRAMP5* and *-6* were at chromosome 10 (Figure 2). The selection pressure acting on *NRAMP* genes was inferred from the ratio of non-synonymous (Ka) to synonymous (Ks) substitution values, and our data indicated that all the *PvNRAMPs* were under evolutionary pressure showing on average a 1.5 Ka/Ks ratio. Additionally, the gene structures were compared among the *PvNRAMPs*. The organization of introns and exons indicated that NRAMP genes could be divided into two types (Figure 3). Type I was formed by 13 short exons with lengths ranging from 79 to 342 bp and intron sizes of 76 bp to 1018 bp. This group included *PvNRAMP*1, -6, and -7, with an average identity of approximately 86% at some significant sequences, with the highest identity equal to 100% and the lowest equal to 75%. In addition, the fifth exon in *PvNRAMP1* had similarity to two exons in *PvNRAMP6*, meaning that this exon was split into two coding sequence at the fifth and seventh exon position in *PvNRAMP6*. In another type formed by *PvNRAMP4*, *-3*, *-2,* and *-5*, the exon numbers were restricted to four, with high similarities among them and with exon lengths from 137 bp to 653 bp and intron lengths of 91 bp to 1540 bp. To investigate if such exon-intron organization was conserved among *NRAMPs*, we analyzed 20 homologs in *G. max*, *M. truncatula*, *A. thaliana* and *O. sativa* (Figure S1). *AtNRAMP1* and *-6* from *Arabidopsis* displayed 11 and 13 exons, respectively. *OsNRAMP1*, *-3*, *-4,* and *-5* from rice and *MtNRAMP1*, *-2,* and *-3* from *M. truncatula* showed 13 exons with limited size variation, while intron size varied largely (Figure S1). Type II grouped *AtNRAMP2*, *-3*, *-4,* and *-5* from *Arabidopsis*, and the rice genes *OsNRAMP2* and *-6*, *MtNRAMP4*, *-5*, *-6,* and *-7* from *M. truncatula,* and *GmDMT1* from *G. max* displayed preserved number and size of exons/introns (Figure S1). Our results indicated that two types of gene structure were conserved among *NRAMP* homologs.

### Phylogenetic comparison of *PvNRAMP* genes with their homologs in *A. thaliana*, *O. sativa*, *G. max* and *M. truncatula*

**Figure 4:**
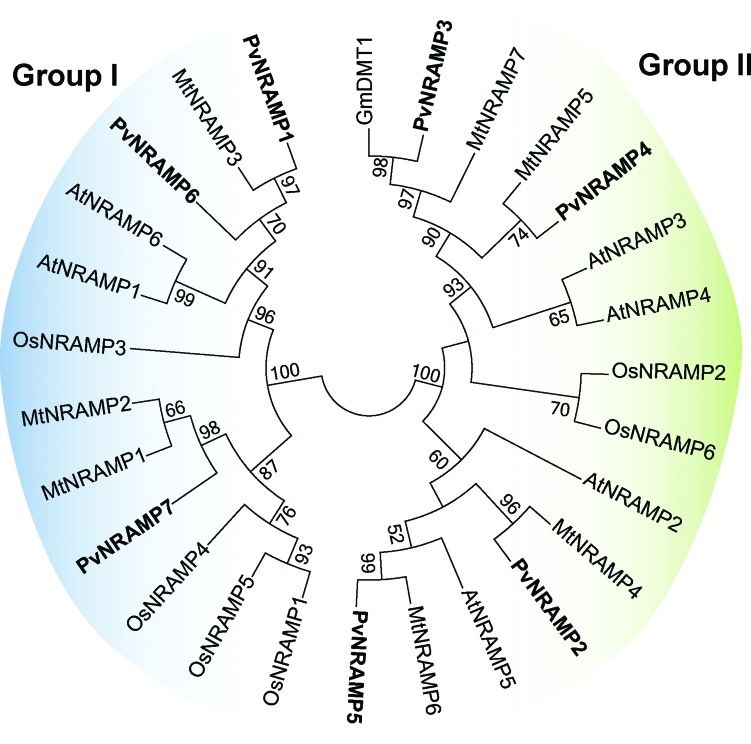
Phylogenetic tree of NRAMP protein sequences. The unrooted tree was generated based on the Maximum Likelihood statistical method and the numbers at the internal node represent the percentage of 10,000 replicates in which the sequences are grouped in the bootstrap test. The different colors indicate the groups. The PvNRAMP protein sequences are in bold: PvNRAMP1 (Phvul.005G182000); PvNRAMP2 (Phvul.009G069700); PvNRAMP3 (Phvul.003G238600); PvNRAMP4 (Phvul.002G014300); PvNRAMP5 (Phvul.010G110500); and PvNRAMP7 (Phvul.009G127900). MtNRAMP1-MtNRAMP7 (*Medtr3g088460, Medtr3g088440, Medtr2g104990, Medtr3g102620, Medtr5g016270, Medtr8g028050*, and *Medtr4g095075*, respectively) and representative plant NRAMP homologues: AtNRAMP1-7 (*At1g80830*, *At1g47240*, *At2g23150*, *At5g67330*, *At4g18790*, and *At1g15960*, respectively) and OsNRAMP1-6 (*Os07g0258400*, *Os03g0208500*, *Os06g0676000*, *Os01g0503400*, *Os07g0257200*, and *Os12g0581600*, respectively). The TjNRAMP4 (Q7XB56), TcNRAMP4 (DQ418489), TcNRAMP3 (EF639294), and GmDMT1 (*Glyma17g18010*).

**Figure 5:**
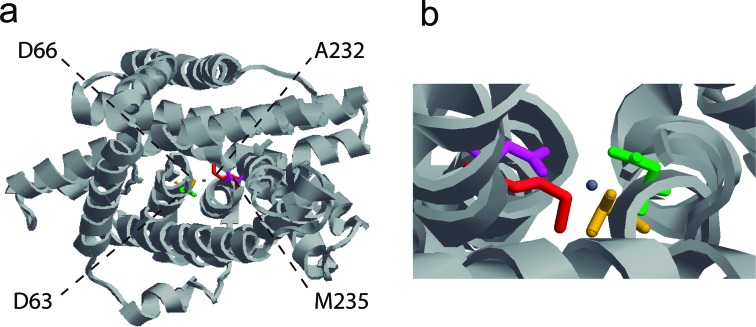
Predicted 3D structure of NRAMP transporter. Tertiary structure of PvNRAMP7 predicted by SWISS-MODEL software based on the template S. capitis DMT1 (ID: 4wgw.1). (a) The ribbon structure is shown in gray, the amino acids coordinating with manganese (II) ion (shown in blue) are illustrated in different colors: D60 (green); N63 (orange); A232 (red); and M235 (pink). (b) The details of metal coordinating amino acids are displayed.

To explore the phylogenetic association among NRAMP homologs in plant genomes, we generated a phylogenetic tree based on putative amino acid sequences of 30 NRAMPs from *Arabidopsis,* rice (*Oryza sativa*), *M. truncatula* and common bean. The tree topology indicated separation into two distinct groups (Figure 4). Each group contained sequences from different species, which suggests a close genetic conservation among them. Group I consisted of 12 members and their protein alignment showed that 26% of the amino acids were identical and localized at conserved positions. This group included previously characterized cation transporters, including AtNRAMP1 and OsNRAMP1, that are involved in the maintenance of iron homeostasis in Arabidopsis and rice, respectively (Curie *et al.*, 2000; Takahashi *et al.*, 2011), and the importer MtNRAMP1 that is required for the iron and manganese uptake by rhizobia-infected nodules in *M. truncatula* (Tejada-Jiménez *et al.*, 2015). In several organisms, the absorption of manganese and iron occurs in an interdependent manner, using the same transport proteins (Fitsanakis *et al.*, 2010). According to our data, the closest homolog of MtNRAMP1 in common bean was *PvNRAMP7* (Figure 4) with 83% of shared amino acid identity. To investigate its association with metal transportation, the tertiary structure was predicted using bioinformatics tools. The *PvNRAMP7* structure was modeled using *Staphylococcus capitis* DMT as template (Ehrnstorfer *et al.*, 2014) (Figure 5a). The quality of property combinations between target and template was estimated by GMQE (Global Model Quality Estimation), resulting in a score of 0.56. The predicted structure showed 12 transmembrane domains (Figure 5a) and potential Mg-coordinating amino acids at the positions Asp60, Asn63, Ala232 and Met235 (Figure 5b). Results analogous to those found in MtNRAMP1 (Tejada-Jiménez *et al.*, 2015) were observed for *PvNRAMP1* (Figure S2a) and *-6* (Figure S2b), which shared 58% of amino acid identity with MtNRAMP1. Therefore, our data suggest that these genes might play a role in iron homeostasis and potentially also for manganese transport in *P. vulgaris*. Group II consisted of 18 sequences, sharing 38.2% of identical amino acids located at conserved positions across the members. This group included *PvNRAMP3* and *-4*, which were grouped with the GmDMT1 (Figure 4), a transporter from soybean involved in the nodulation process (Kaiser *et al.*, 2003). *PvNRAMP5* was closer to the transporter AtNRAMP5 (Figure 4) that negatively responds to higher concentrations of iron in flowers (Ravet *et al.*, 2009). *PvNRAMP2* and -*4* were placed at the same branch as MtNRAMP4 and -5 (Figure 4), respectively, the functions of which have not yet been characterized. None of the *PvNRAMP* sequences showed enough affinity to the vacuolar transporters AtNRAMP3 and -4.

### Expression analysis of *PvNRAMP* genes during common bean development

**Figure 6:**
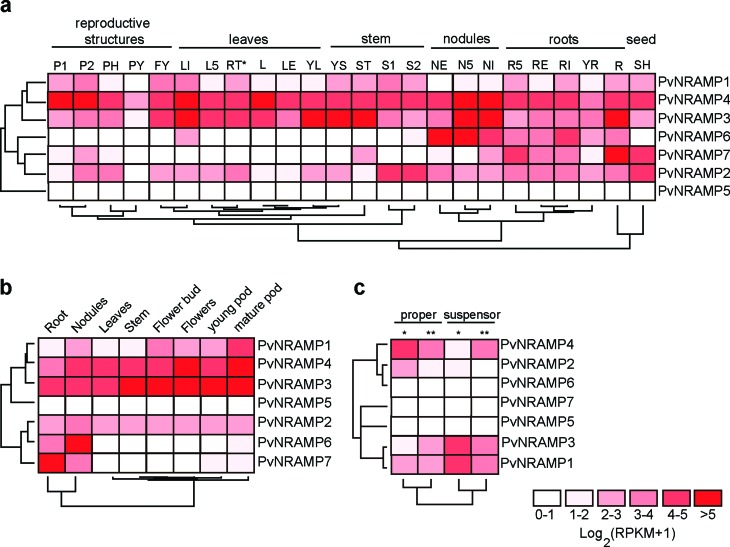
Expression levels of *P. vulgaris* NRAMP genes. Heatmaps showing the hierarchical clustering of NRAMP family genes grouped according to the expression patterns of RNA-Seq data collected from different tissues during the development of *P. vulgaris*. Gradient color ranging from white to bright red corresponds to expression values calculated as Log2(RPKM + 1), as described by the legend at the bottom. (a) RNA-Seq based on gene expression atlas of the common bean (O’Rourke *et al.*, 2014). Expression P1: pods with 10–11 cm; P2: pods with 12–13 cm; PH: pods with 9 cm; PY: young pod; FY: young flower; LI: leaf tissue after 21 days from inoculation of an ineffective rhizobium, L5: leaf tissue 5 days after the inoculation of an effective rhizobium; RT: root tips; SH: heart stage seeds; L: leaf tissue of non-inoculated plant; LE: leaf tissue 21 days after the inoculation of an effective rhizobium; YL: young leaf; YS: young stem; ST: shoot apical meristem; S1: seeds with 6–7 mm; S2: seeds with 8–10 mm; NE: effectively fixing nodules; N5: pre-fixing nodules collected at 5 days after inoculation; NI: ineffective nodules; R5: root system separated from pre-fixing nodules; RE: root separated from effectively fixing nodules; RI: root separated from ineffectively fixing nodules; YR: radicle; R: roots without rhizobium inoculation; RT: root tips; SH: heart stage seeds. (b) 10-day-old root and stem tissues and pool of leaves at different developmental stages (Schmutz *et al.*, 2014). (c) Embryo dissected by laser at heart stage (GSE57535), the expression data in the proper and suspensor embryo tissues are shown. (*) and (**) asterisks correspond to first and second biological replicates.

To gain further insights into biological function of *PvNRAMPs*, their expression levels were investigated using available RNA-Seq data retrieved from different common bean tissues across developmental processes. In Group I, *PvNRAMP3* and *-4* shared a similar expression pattern, indicating that both were recruited during all stages of common bean development. The expression levels of *PvNRAMP2* also exhibited expression in distinct tissues; nevertheless, the levels were lower compared with *PvNRAMP3, -4,* and *-5* (Figure. 6a-c). For Group II, the *PvNRAMP6* and *-7* genes were preferentially expressed in the root system, with the highest expression of *PvNRAMP6* in active nodules (Figure 6a, b). *PvNRAMP1* showed higher expression in reproductive structures, including seeds (Figure 6b, c). At early stages of common bean development, *PvNRAMP3* and *PvNRAMP4* were differentially up-regulated. *PvNRAMP3* was mainly recruited in the suspensor, an embryonic region formed by few cells that connect the embryo to the surrounding endosperm (Kawashima and Goldberg, 2010), while *PvNRAMP4* was preferably expressed in the embryo proper (Figure 6c). *PvNRAMP5*, *-6,* and *-7* were not expressed (Figure 6c) at this stage of bean ontogenesis. Therefore, the genes from Group I showed a broad expression across different plant tissues (Figure 6a-c). In contrast, the transporters from Group II exhibited an expression pattern limited to certain tissues.

For further comparisons, the expression levels of the *NRAMP* homologs in other Fabaceae species were obtained (Figures S3, S4). Analysis of the gene expression atlas of *G. max* (Qin *et al.*, 2017) and *M. truncatula* (Benedito *et al.*, 2008) indicated that besides *GmDMT1* and *MtNRAMP1*, essential genes for nodulation (Kaiser *et al.*, 2003)(Tejada-Jiménez *et al.*, 2015), *GmNRAMP7* (Figure S4) (Qin *et al.*, 2017) and *MtNRAMP2* (Figure S3a) were highly expressed during the root interaction with rhizobia. Comparing different tissues at distinct developmental stages, *MtNRAMP2* showed an expression level close to *MtNRAMP1* (Figure S3a). Investigation of micro-dissected tissues indicated that the biological function of *MtNRAMP2* relied on surrounding tissues of rhizobia-infected and nematode-infected cells (Figure S3b). In the phylogenetic branch of GmNRAMP7, MtNRAMP1 and -2 were closest to PvNRAMP7 (Figure S5), that was seen up-regulated in active nodules (Figure 6b and Figure S4). Similar expression patterns were found for *PvNRAMP6* (Figure 6b) and for its homologs *GmNRAMP5a* and *GmNRAMP5b* (Figure S4, S5). From Group I, the *PvNRAMP5* showed weak expression signal in several tissues (Figure 6a), similar to its homolog in soybean (Figure S4). Expression levels in meristematic, vegetative, and reproductive structures of corresponding homologs of *PvNRAMP1*, *-2*, *-3*, *-4,* and *-5* in *M. truncatula* (Figure S3a, S5) and *G. max* (Figures S4, S5) were also compared. Analogous to these NRAMP genes in common bean (Figure 6a, b), the homologs in other Fabaceae plants showed similar transcriptional patterns during plant development (Figures S3, S4), including the nodule-essential gene *GmDMT1* which was preferentially expressed in root hairs and nodules compared with other tissues (Figure S4). Taken together, our data indicated that the biological functions of these genes are highly conserved among the three species.

### Transcriptional regulatory elements in *PvNRAMP* genes

To get further insights into the putative biological function of *NRAMP* genes in *P. vulgaris*, regulatory *cis*-elements located in the promoter and untranslated regions (UTR) were investigated. First, we searched for Iron Responsive Elements (IREs) motifs in the NRAMP transcriptional sequences. One single IRE regulatory motif (AG TTGTTCATTCAGAGAGTTAGGTAATCAAT) was found in the 3’UTR region of *PvNRAMP3*, starting at 1584bp–1615bp. The GmDMT1, an IRE-contained soybean gene (Kaiser *et al.*, 2003), and PvNRAMP3 were placed at the same branch (Figure S5). To obtain further information of transcriptional regulation of PvNRAMPs, we investigated the presence of regulatory elements in their promoter regions. Regulatory *cis*-elements of each putative *PvNRAMP* promoter sequences were identified using the PlantCARE program (Table 2). We observed that the drought- and light-responsive regulatory elements were present in all sequences. The regulatory elements associated with Methyl Jasmonate (MeJa) were limited to the promotors of *PvNRAMP2*, *-3,* and *-6*, whereas the upstream sequences of *PvNRAMP1* and *-7* showed elements responsive to fungal elicitors. We also observed that all analyzed regions exhibited at least one regulatory element associated with regulation and maintenance of biological processes like hormonal responses. Among them, *PvNRAMP7* is the only gene in which the transcriptional response might be regulated by abscisic acid. These results indicated that the regulation of *PvNRAMP* transcriptional activity may be activated during the plant development and by external biotic and abiotic stresses.

**Table 2:** List of regulatory *cis*-elements identified in the putative promoter regions of seven *NRAMP* genes in *P. vulgaris*.

Putative biological response		NRAMP1	NRAMP2	NRAMP3	NRAMP4	NRAMP5	NRAMP6	NRAMP7
Abiotic stress	Light	4	6	7	5	4	6	3
	Drought	1	1	1	1	1	1	1
	Heat/cold	-	-	1	1	1	1	-
Biotic stress	MeJa	-	2	2	-	-	-	-
	SA/Defense	2	2	2	2	2	2	2
	Fungal elicitor	1	-	-	-	-	-	1
Hormonal response, growth and development	Gibberellin	1	-	1	-	1	-	1
	Abscisic acid	1	1	1	1	1	1	-
	Ethylene	1	-	-	-	-	-	-
	Cell cycle	-	1	-	-	-	-	-
	Endosperm	-	1	2	2	1	2	-
	Meristem	-	-	-	1	-	1	1
	Leaf	-	-	-	-	-	-	2
	Seed	-	-	-	-	-	1	-

### Expression of *PvNRAMP* genes in response to *Colletotrichum lindemuthianum infection*

To further investigate the potential involvement of *PvNRAMP* genes in biotic stress, as suggested by the analysis of regulatory elements, we infected common bean leaves with the pathogenic fungus *Colletotrichum lindemuthianum.* The transcriptional response of *NRAMP* genes showed that in general the transporters were down-regulated following *C. lindemuthianum* infection (Table 3). We observed strong suppression with statistical support for *PvNRAMP1*, *-2* and *-3*, while no amplification was observed for the *PvNRAMP7* and -*5* genes (Table 3). These data suggested a potential interruption of NRAMP-mediated transport in common bean during the pathogen invasion.

**Table 3:** Quantitative RT-PCR for *PvNRAMPs* in *C. lindemuthianum-*infected leaves.

Name	qRT-PCR (fold change)	*p*-value
*PvNRAMP1*	-3.34	0.001
*PvNRAMP6*	-2.42	0.0615
*PvNRAMP7*	No amplification	-
*PvNRAMP2*	-10.57	0.001
*PvNRAMP3*	-5.43	0.0385
*PvNRAMP4*	-1.01	0.861
*PvNRAMP5*	No amplification	-

* Fold change means the gene expression levels in infected leaves divided by the values in non-infected tissue

## Discussion

Common bean is an important source of nutrients in developing countries. It provides critical metals for human health, such as iron, manganese, zinc, etc. (Beebe *et al.*, 2000). Thus, understanding the mechanisms involved in the accumulation and transport of metals in common bean tissues is relevant for food security. Previous studies have demonstrated the relevance of the NRAMP carrier family to maintain metal homeostasis in Arabidopsis, rice, and *M. truncatula* (Sebastien and Schroeder, 2004). However, detailed information on this transport family in the common bean was missing. Here, we used a bioinformatics approach to identify the members of NRAMP family in the *P. vulgaris* genome and to determine their biological role during common bean development.

## There are two groups of plant NRAMPs that differed in their origins

The MntH/NRAMP family metal transport emerged as an adaptation to the oxygen-rich environment, and this family is found in the genomes of eukaryotes and prokaryotes (Cellier *et al.*, 1996). The key characteristic of this transporters is the presence of the bacterial Consensus Transport Sequence in the core of the preserved hydrophobic pocket (Cellier *et al.*, 1995). The protein structure reflects the preserved biological role of MntH/NRAMP that conveys the control of the redox metal concentration (Cellier *et al.*, 2001). In plants, the differences seen among members of the NRAMP family raised the hypotheses that it is a polyphyletic group (Sebastien and Schroeder, 2004). The first group (Group I) has a putative primary amino acid sequence that is apparently closer to MntH/NRAMP from bacteria, whereas the second group (Group II) is more related to those from animals (Sebastien and Schroeder, 2004). Indeed, our phylogenetic data and comparison of protein features, gene structure, and expression patterns are in support of the hypothesis of distinct origins. Similar to AtNRAMP3, -4 in *A. thaliana* (Lanquar *et al.*, 2005 *,* 2010) and GmNRAMP1a, 2a, 2b and 3a in soybean (Qin *et al.*, 2017), the bacteria-like PvNRAMPs showed a potential function at membranous organelles (Table 1). The putative proteins showed a basic IP, which differed from the animal-like group that showed an acidic isoelectric point (Table 1). Consistently, the fragmented and numerous short exons sequences from Group I contrasted with the concise gene structure seen in Group II (Figure 3 and S1). Additionally, the expression patterns of the NRAMP homologs in Fabaceae also differed between the groups. The stable expression across multiple plant organs suggested that genes from Group II may serve to maintain the biological role in basic cellular processes, transporting metals for growing tissues when they are necessary. In contrast, the genes from Group I showed a higher expression that was generally limited to certain tissues, suggesting a more specific function during plant development. Therefore, our data indicated that NRAMP sequences in plants may be divided into two groups that diverged early during evolution.

## Potential role of NRAMP genes in *P. vulgaris*

Interactions between different organisms are often accompanied by morphological changes, including the formation of specialized organs. For example, parasitic knot nematodes provoke the reorganization of root compartments to form giant multinuclear cells at the nematode feeding sites (Jones, 1981). Similarly, symbiotic interactions between nitrogen-fixing bacteria and leguminous plants result in the development of root structures called nodules (Masson-Boivin *et al.*, 2009), which require an intense iron mobilization as cofactor of critical enzymes involved in nodulation (Tang *et al.*, 1990). During nodule formation, when drastic morphological changes occur, the surrounding cells harbor transporter proteins to fully supply the newly-formed organ with necessary nutrients. The up-regulation of *MtNRAMP2* limited to the region that is circumjacent to the rhizobia-infecting zone and root galls (Figure S3) suggests a conserved function of this protein as a carrier to supply minerals for plant-interacting organisms throughout their life cycle. The closest gene to *MtNRAMP2* in amino acid sequence is *MtNRAMP1*, which is recruited for both iron and manganese uptake for nodulation in *M. truncatula* (Tejada-Jiménez *et al.*, 2015). Its homolog in soybean, *GmNRAMP7* (Figure S5) gene is highly regulated during nodulation (Figure S4), activated upon iron starvation and suppressed under iron toxicity (Qin *et al.*, 2017). Our data comparison indicated that orthologous pairs among Fabaceae maintained similar expression patterns, suggesting a potential conserved biological function. The predicted tertiary structure of *PvNRAMP7* and *-6* (Figures 5, S2), their high similarities with the homologs *MtNRAMP1* (Figures S3, S5) and *GmNRAMP7* (Figures S4, S5) in terms of expression patterns allowed us to infer that *PvNRAMP7* and *-6* might be the NRAMP transporters used for iron/manganese trafficking involved in initiating the nodules in *P. vulgaris*.

Plants adopted two strategies for iron uptake from the environment (Marschner and Römheld, 1994). The first strategy, found in all the plants except Poaceae, is that root cells release into the rhizosphere substances that reduce the iron forms so that they can be absorbed by carrier proteins, such as AtNRAMP1 ([Bibr B21] Curie *et al.*, 2000), MtNRAMP1 (Tejada-Jiménez *et al.*, 2015) and GmDMT1 (Kaiser *et al.*, 2003). The second strategy is followed by the Poaceae family, which release into the soil the Fe-chelating molecules known as siderophores, where specific transporters facilitate the entry of the ferric-iron-siderophores complex into the plant cell (Marschner and Römheld, 1994).

Most genes encoding iron transporters harbor an IRE motif that plays a direct role in iron regulation (Muckenthaler *et al.*, 2008). Briefly, under iron deficiency, the IRE motif becomes an available binding site for iron-regulating proteins (IRPs) responsible for stabilizing the mRNA, allowing an increase in the transcriptional level (Muckenthaler *et al.*, 2008). Several iron-regulated plant genes contain IRE motifs at their UTRs (Petit *et al.*, 2001; Vert *et al.*, 2001, 2002), although the role of the IRE/IRP mechanism in iron homeostasis in plants is controversial (Arnaud *et al.*, 2007). The iron-induced *GmDMT1* gene encoding an NRAMP transporter shows a single IRE motif in its 3’UTR, and it is recruited to absorb iron into soybean root nodules (Kaiser *et al.*, 2003). In *P. vulgaris*, only the *PvNRAMP3* transcript showed a single IRE motif in its 3’UTR, associating this gene with iron metabolism in beans. The expression spectrum ranging from embryo to mature pods (Figure 6a-c), including the nodules, indicated that this function is widely required and that it is not limited to the plant-rhizobia interaction.

In the early stages of plant development, embryo growth is one of the critical events in which the metal transporters are intensively required (Kawashima and Goldberg, 2010). During the embryo formation, a set of cells called suspensor maintains the embryo connected to the surrounding tissue. The basic function of the suspensor is to transport necessary metabolites to embryo proper. The differential transcriptional regulation of NRAMP genes in *P. vulgaris* indicated that three genes are recruited during the early stages of bean formation (Figure 6c). *PvNRAMP4* is up-regulated in suspensor cells and might facilitate the regulation of the concentration of divalent metals from endosperm to embryo, while *PvNRAMP1* and *-3* might be acting in the embryo proper to allocate the metals to needed cell compartments.

Besides the recruitment of NRAMP transporters during plant development and host-microorganism interactions, members of this family actively participate in the accumulation of toxic metals in plants (Thomine *et al.*, 2000; Mizuno *et al.*, 2005; Oomen *et al.*, 2009). The pollution in water bodies and river tributaries that supply agricultural fields caused increased concern over the accumulation of toxic metals in crop cultivation (Clemens and Ma, 2016). OsNRAMP1 is directly associated with arsenic (As) and cadmium (Cd) uptake in rice (Takahashi *et al.*, 2011; Tiwari *et al.*, 2014), while TjNRAMP4, TcNRAMP3 and -4 are recruited in the heavy-metal-tolerant species *T. japonicum* (Mizuno *et al.*, 2005) and *T. caerulescens* (Oomen *et al.*, 2009). In the common bean, few studies have investigated the impact of metal pollutants in plant metabolism. Thus, the identification and characterization of *PvNRAMP* transporters provided in this study may help us to elucidate the involvement of these proteins in toxic metal uptake and accumulation in beans.

The fungal pathogen *C. lindemuthianum* is the causal agent of anthracnose disease, which strongly affects bean productivity (Couto *et al.*, 2005; Aragão *et al.*, 2011). This pathogen adopted a hemibiotrophic lifestyle, varying from an early biotrophic phase to a necrotrophic phase (Perfect *et al.*, 1999). During the biotrophic stage, the pathogenic fungus cohabitating with living plant cells absorbs nutrients from the host (Perfect *et al.*, 1999; Gan *et al.*, 2013). In vertebrates, one of the host strategies to block the pathogenic progress is to restrict the availability of essential metals such as iron, zinc, manganese and copper (Kehl-Fie and Skaar, 2010). Thus, to withhold the pathogenic growth, the host transcriptional machinery suppresses the expression of the metal NRAMP1 transporter (Wessling-Resnick, 2015). This nutritional immunity strategy through metal retention found in vertebrates might also occur in plants. The modulation of two isoforms of OsNRAMP6 by a microRNA during *Magnaporthe oryzae* infection was described as contributing to disease resistance (Campo *et al.*, 2013; Peris-Peris *et al.*, 2017). Here, we observed that three members of the NRAMP family in *P. vulgaris* (*PvNRAMP1, -2* and *-3*) were suppressed upon *C. lindemuthianum* invasion during its biotrophic stage (Table 3). This data may indicate that PvNRAMP transporters may play a role in *P. vulgaris* as a defense strategy, retaining the accessibility of metals essential for the pathogenic growth, thereby restricting disease advance in plant tissues.

## Conclusions

Common bean has seven members of the NRAMP family. The phylogenetic analysis using sequences from representative species indicated that there are two NRAMP groups in *P. vulgaris*. An expression pattern comparison of *NRAMP* genes in Fabaceae from multiple tissues at different developmental stages indicated a conserved biological function among NRAMP homologs. The transcriptional level analysis revealed that *PvNRAMP6* and *-7* may have a role during symbiosis with beneficial microorganism, whereas *PvNRAMP1, -2, -3, -4 and -5* might be required for general metal homeostasis during all developmental stages of the common bean. The qRT-PCR analysis indicated that *NRAMP* genes are not required during infection by the pathogen *C. lindemuthianum*. Taken together, the systematic genome-wide-analysis of *NRAMP* genes in the *P. vulgaris* genome, supplies basic information on their role in regulating metal homeostasis during development and interaction with microorganisms in common bean.

## Supplementary Material

The following online material is available for this article:

Figure S1 - Schematic structure of *NRAMP* genes in *P. vulgaris, M. truncatula, A. thaliana, O. sativa* and *G. max*.

Figure S2 - Putative 3D structure of NRAMP transporter.

Figure S3 - Expression levels of *NRAMP* genes in *M. truncatula.*

Figure S4 - The expression of NRAMP genes in vegetative and reproductive tissues in soybean.

Figure S5 - Phylogenetic tree of NRAMP genes in Fabaceae.

Table S1 - Gene specific primers for *P. vulgaris NRAMP* genes.
