# Supplementary material for: Genome-wide characterization of the NRAMP gene family in Phaseolus vulgaris provides insights into functional implications during common bean development
Source: Genet Mol Biol. 2018 Oct 11;41(4):820–33. doi: 10.1590/1678-4685-GMB-2017-0272 (PMC6415609; doi:10.1590/1678-4685-GMB-2017-0272)
Supplement: Supplementary file 1 [file 1415-4757-GMB-1678-4685-GMB-2017-0272-s006.pdf]

Supplementary Material to "Genome-wide characterization of the NRAMP gene family in *Phaseolus vulgaris* provides insights into functional implications during common bean development"

Table S1 – Gene specific primers for *P. vulgaris NRAMP* genes used for quantitative real-time PCR.

Gene name	Primer	Sequence (5' to 3')	Length (mer)	Tm (°C)	Amplicon size
PvNRAMP1	PvNRAMP1-qRT-PCR-F	TCACTTCTGCACCCTCTTCATC	22	60	134 bp
	PvNRAMP1-qRT-PCR-R	AAAAGCTTCGGTTGCCAGTG	20	59.6	
PvNRAMP2	PvNRAMP2-qRT-PCR-F	ACGCCGGAAAGTTTGATCAG	20	58.8	147 bp
	PvNRAMP2-qRT-PCR-R	CGCTTCGTATGCCACATCTTC	21	59.7	
PvNRAMP3	PvNRAMP3-qRT-PCR-F	TCAACCTCTCTTCTCACTGCAC	22	60	124 bp
	PvNRAMP3-qRT-PCR-R	GGATTCGTAGGCTGTTTCTTGC	22	59.9	
PvNRAMP4	PvNRAMP4-qRT-PCR-F	TGGGCAGTTTCAGAATTGGC	20	59	121 bp
	PvNRAMP4-qRT-PCR-R	ACTGCTCCATTCACTTCAGAGG	22	60	
PvNRAMP5	PvNRAMP5-qRT-PCR-F	TCCCCCTTCTTACTTTGGTGTC	22	59.6	101 bp
	PvNRAMP5-qRT-PCR-R	TATTACCAGCACAGCCACACTC	22	60.4	
PvNRAMP6	PvNRAMP6-qRT-PCR-F	GCGCCGTTGATTGAGAATTC	20	58.2	86 bp
	PvNRAMP6-qRT-PCR-R	GGCCCCACGTATGCAAATAAG	21	59.7	
PvNRAMP7	PvNRAMP7-qRT-PCR-F	TAGCTGAGCTCGCTGTTATAGC	22	60	70 bp
	PvNRAMP7-qRT-PCR-R	AGTATGTTCAACGCGAAGGC	20	58.9	
