# Supplementary material for: Genome-wide characterization of the NRAMP gene family in Phaseolus vulgaris provides insights into functional implications during common bean development
Source: Genet Mol Biol. 2018 Oct 11;41(4):820–33. doi: 10.1590/1678-4685-GMB-2017-0272 (PMC6415609; doi:10.1590/1678-4685-GMB-2017-0272)
Supplement: Supplementary file 2 [file 1415-4757-GMB-1678-4685-GMB-2017-0272-s001.pdf]

Supplementary Material to "Genome-wide characterization of the NRAMP gene family in *Phaseolus vulgaris* provides insights into functional implications during common bean development"

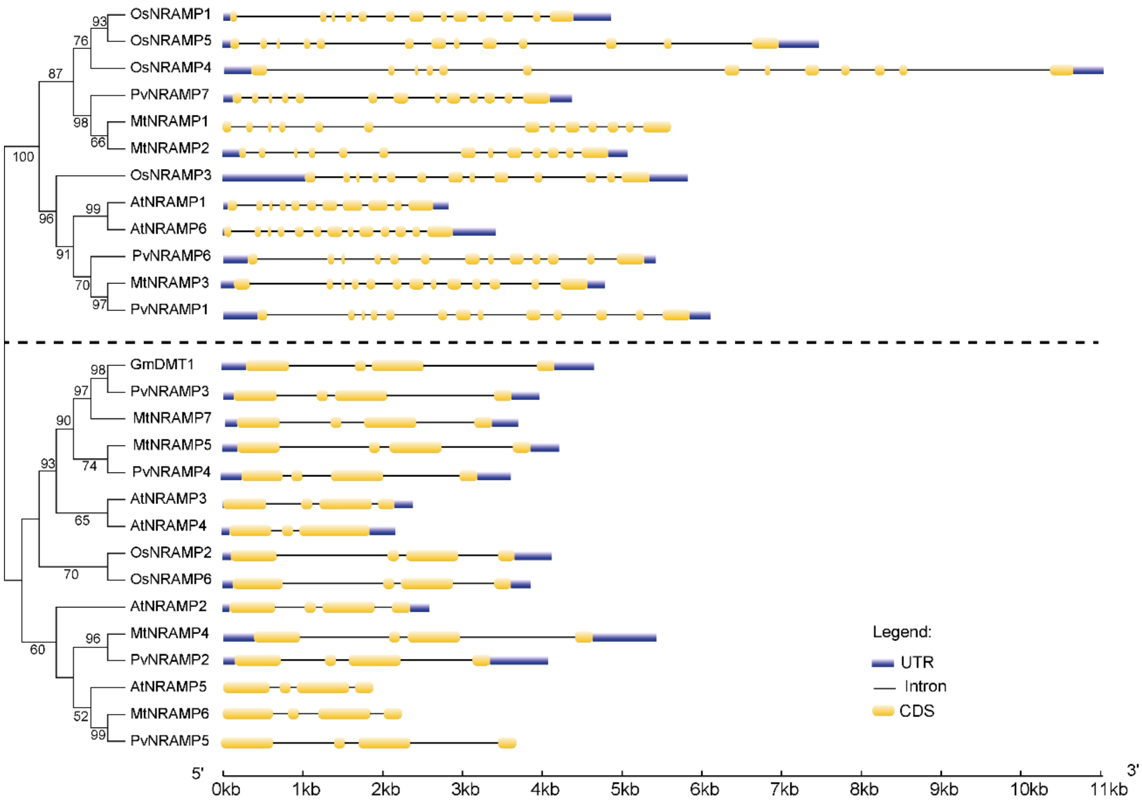

Figure S1 - Schematic structure of *NRAMP* in *P. vulgaris*, *M. truncatula*, *A. thaliana*, *O. sativa* and *G. max*. Phylogenetic tree with introns, exons and UTRs. Lines connected to similar exons and different colors to indicate novel exons. The phylogenetic tree was generated based on the Maximum Likelihood statistical method and the numbers at internal node represent the percentage of 1,000 replicates in which the sequences grouped in the bootstrap test. PvNRAMP1 (Phvul.005G182000), PvNRAMP2 (Phvul.009G069700), PvNRAMP3 (Phvul.003G238600), PvNRAMP4 (Phvul.002G014300), PvNRAMP5 (Phvul.010G110500), and PvNRAMP7 (Phvul.009G127900). MtNRAMP1-MtNRAMP7 (*Medtr3g088460*, *Medtr3g088440*, *Medtr2g104990*, *Medtr3g102620*, *Medtr5g016270*, *Medtr8g028050*, and *Medtr4g095075*, respectively) and representative plant NRAMP homologues: AtNRAMP1-7 (*At1g80830*, *At1g47240*, *At2g23150*, *At5g67330*, *At4g18790*, and *At1g15960*, respectively) and OsNRAMP1-6 (*Os07g0258400*, *Os03g0208500*, *Os06g0676000*, *Os01g0503400*, *Os07g0257200*, and *Os12g0581600*, respectively; GmDMT1 (*Glyma17g18010*).
