# Supplementary material for: Genome-wide characterization of the NRAMP gene family in Phaseolus vulgaris provides insights into functional implications during common bean development
Source: Genet Mol Biol. 2018 Oct 11;41(4):820–33. doi: 10.1590/1678-4685-GMB-2017-0272 (PMC6415609; doi:10.1590/1678-4685-GMB-2017-0272)
Supplement: Supplementary file 3 [file 1415-4757-GMB-1678-4685-GMB-2017-0272-s002.pdf]

Supplementary Material to "Genome-wide characterization of the NRAMP gene family in *Phaseolus vulgaris* provides insights into functional implications during common bean development"

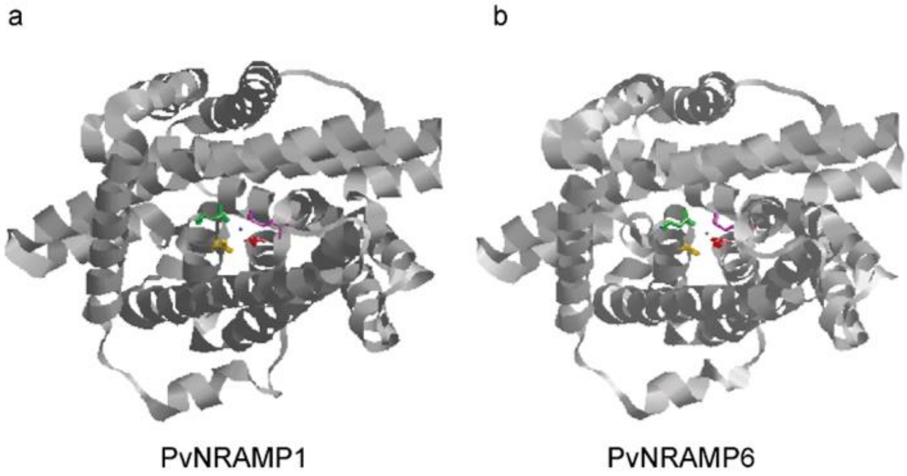

Figure S2 - Putative 3D structure of NRAMP transporter. Tertiary structure predicted by SWISS-MODEL software based on the template *S. capitis* DMT1 (ID: 4wgw.1). The ribbon structure is shown in gray, the amino acids coordinating with manganese (II) ion (shown in blue) were illustrated in different colors: D63 (green), N66 (orange), A235 (red), and M238 (pink). (a) PvNRAMP1. (b) PvNRAMP6.
