# Supplementary material for: Genome-wide characterization of the NRAMP gene family in Phaseolus vulgaris provides insights into functional implications during common bean development
Source: Genet Mol Biol. 2018 Oct 11;41(4):820–33. doi: 10.1590/1678-4685-GMB-2017-0272 (PMC6415609; doi:10.1590/1678-4685-GMB-2017-0272)
Supplement: Supplementary file 4 [file 1415-4757-GMB-1678-4685-GMB-2017-0272-s003.pdf]

Supplementary Material to "Genome-wide characterization of the NRAMP gene family in *Phaseolus vulgaris* provides insights into functional implications during common bean development"

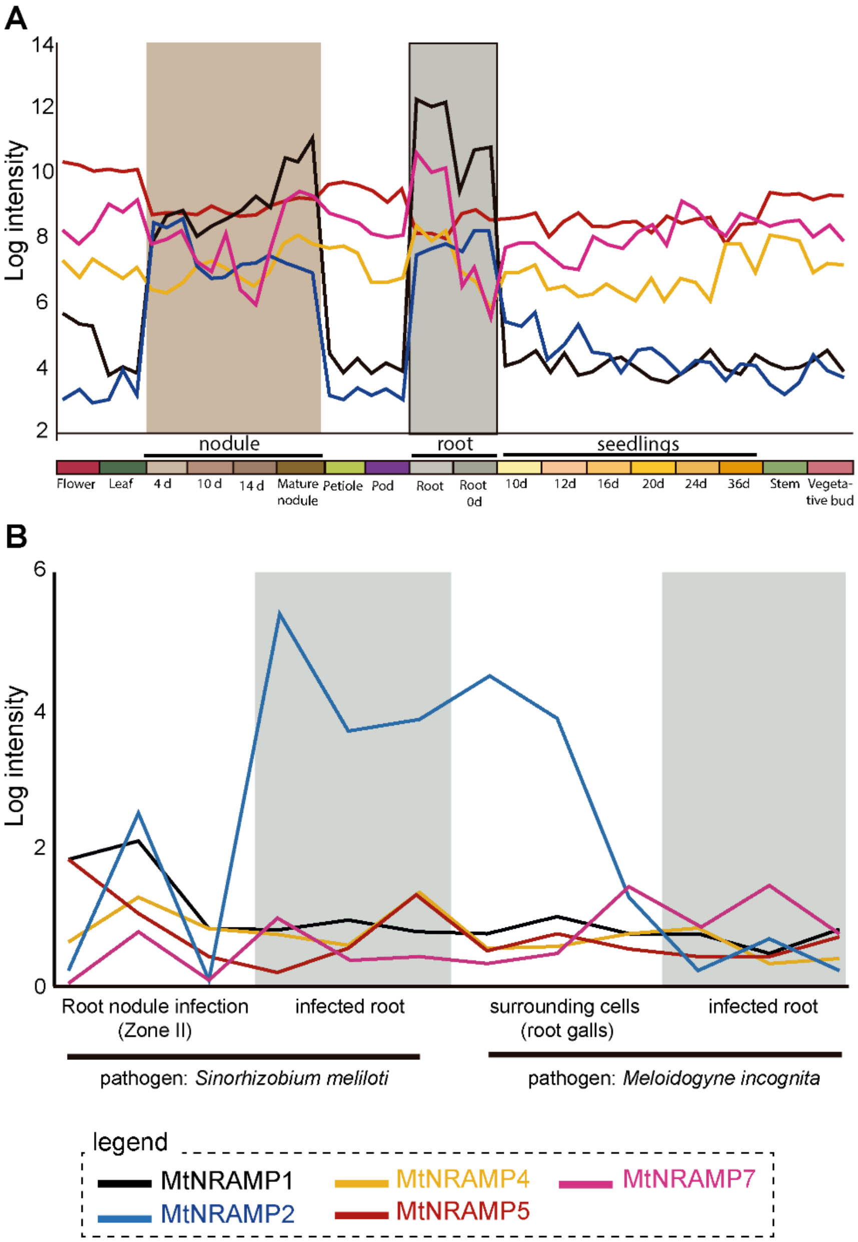

Figure S3 - Expression levels of *NRAMP* genes in *M. truncatula*. Expression values were obtained based on Affymetrix 61K Medicago Genechip. The line colors correspond to expression levels of different NRAMP genes: black for MtNRAMP1 (Probe ID: Msa.2831.1.S1_at); blue for MtNRAMP2 (Mtr.10583.1.S1_at); yellow for MtNRAMP4 (Mtr.42570.1.S1_at); red for MtNRAMP5 (Mtr.40062.1.S1_at); and pink for MtNRAMP7 (Mtr.5938.1.S1_at). In parenthesis is the microarray unique code. The corresponding codes for MtNRAMP3 and -6 were not present in the available data. Each hybridization sample is shown in triplicate. (a) The transcriptional levels corresponding to *M. truncatula* gene expression atlas (Benedito *et al.*, 2008), the pale brown and gray shadows highlight the nodule and root tissues, respectively. (b) Data corresponding to transcriptome of *M. truncatula* interaction with rhizobia *Sinorhizobium meliloti* or the plant parasitic nematode *Meloidogyne incognita* (Damiani *et al.*, 2012). The left panel refers to samples of rhizobia-infected nodule (zone II) and surrounding nodule cells (highlighted by gray shadows). The right panel shows the transcripts from nematode-infected cells (known as giant cells) and the cells neighboring them (highlighted by gray shadows).
