# Supplementary material for: Genome-wide characterization of the NRAMP gene family in Phaseolus vulgaris provides insights into functional implications during common bean development
Source: Genet Mol Biol. 2018 Oct 11;41(4):820–33. doi: 10.1590/1678-4685-GMB-2017-0272 (PMC6415609; doi:10.1590/1678-4685-GMB-2017-0272)
Supplement: Supplementary file 5 [file 1415-4757-GMB-1678-4685-GMB-2017-0272-s004.pdf]

Supplementary Material to "Genome-wide characterization of the NRAMP gene family in *Phaseolus vulgaris* provides insights into functional implications during common bean development"

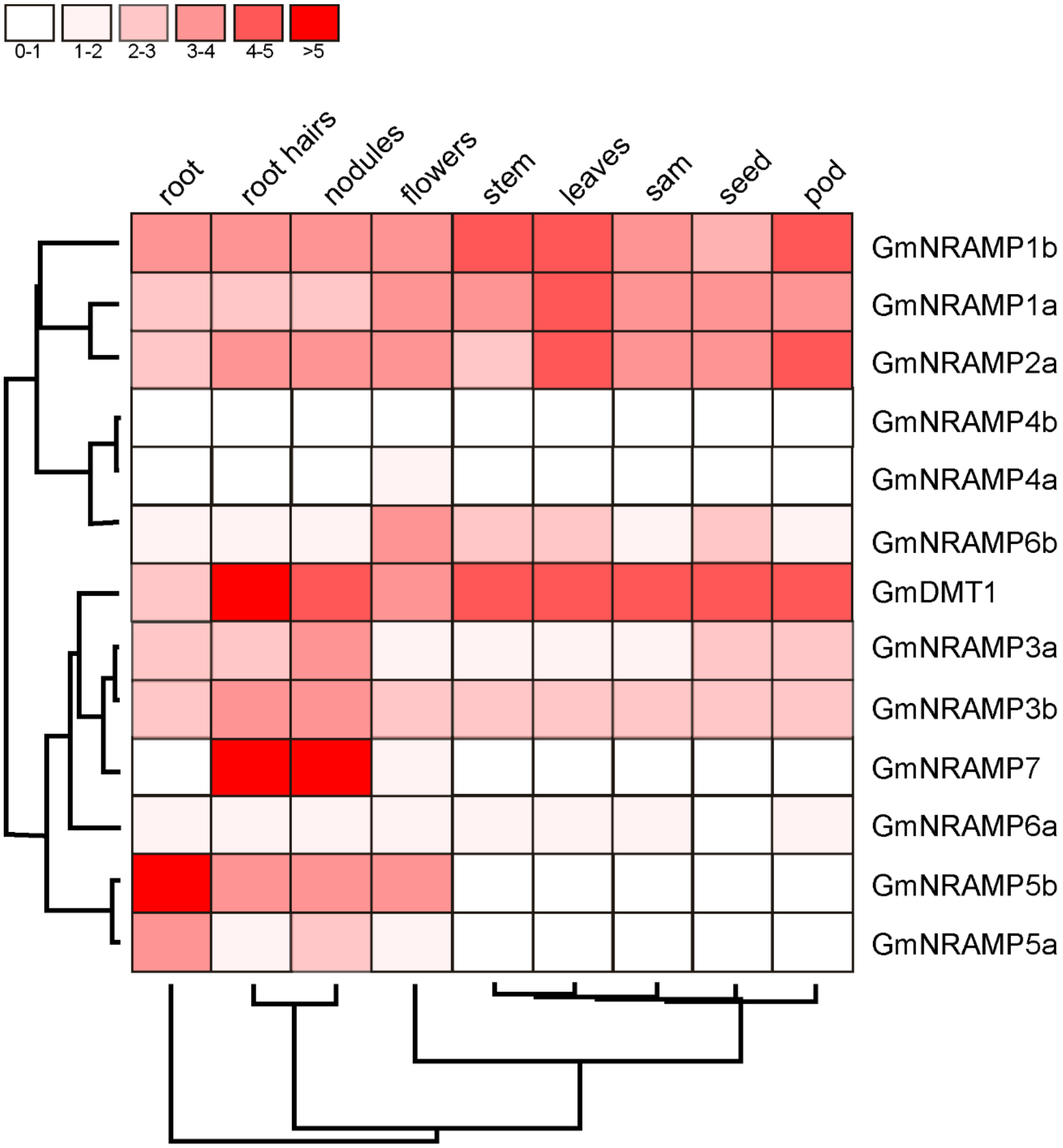

Figure S4 - The expression of NRAMP genes in vegetative and reproductive tissues in soybean. Heatmaps showing the hierarchical clustering of NRAMP family genes grouped according to expression patterns. Gradient color ranging from white to bright red corresponds to expression values calculated as Log2(FPKM + 1), as described by the legend at the top. GmDMT1 corresponds to *Glyma17g18010*.
