# Supplementary material for: Genome-wide characterization of the NRAMP gene family in Phaseolus vulgaris provides insights into functional implications during common bean development
Source: Genet Mol Biol. 2018 Oct 11;41(4):820–33. doi: 10.1590/1678-4685-GMB-2017-0272 (PMC6415609; doi:10.1590/1678-4685-GMB-2017-0272)
Supplement: Supplementary file 6 [file 1415-4757-GMB-1678-4685-GMB-2017-0272-s005.pdf]

Supplementary Material to "Genome-wide characterization of the NRAMP gene family in *Phaseolus vulgaris* provides insights into functional implications during common bean development"

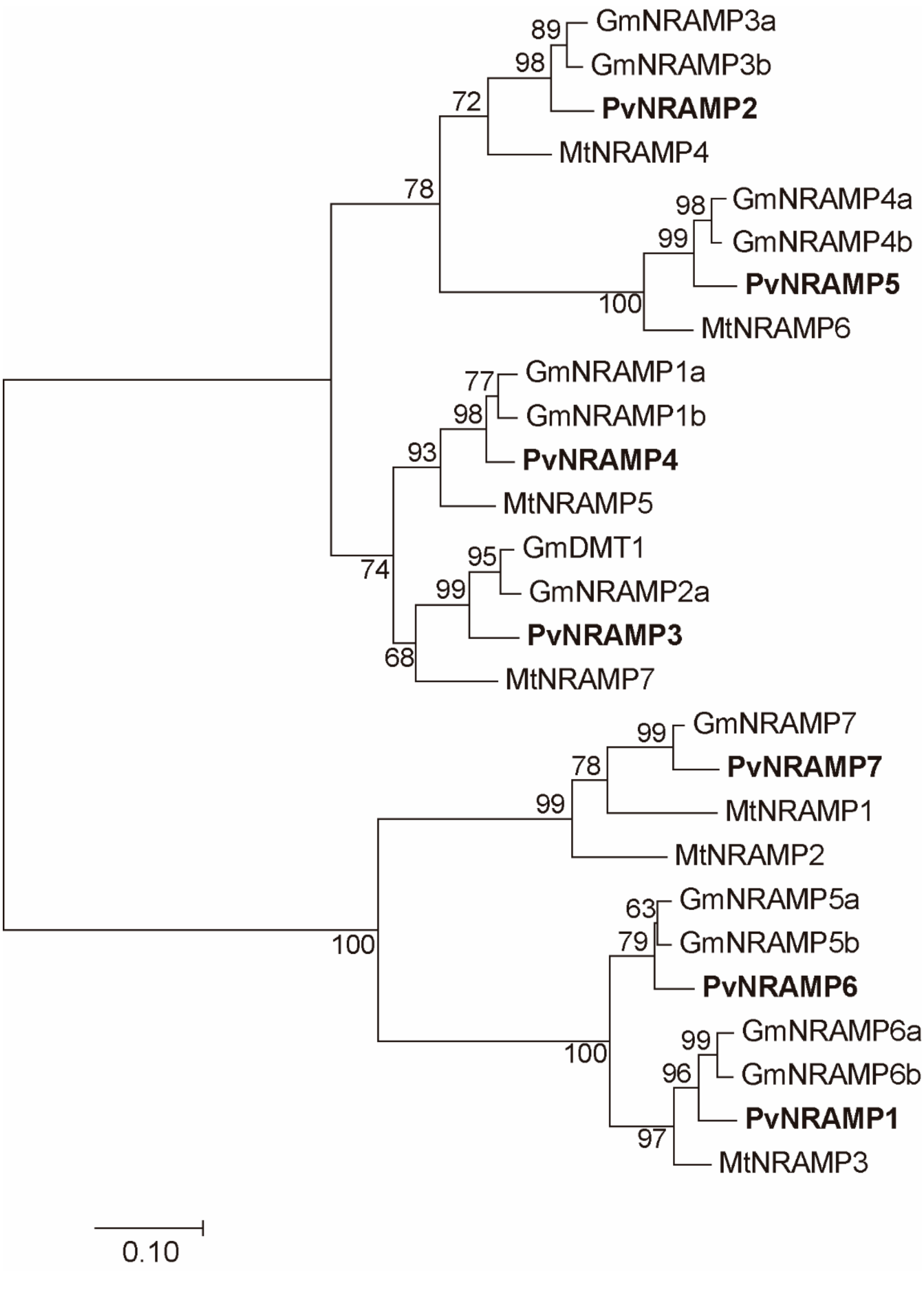

Figure S5 - Phylogenetic tree of NRAMP genes in Fabaceae. The tree was generated based on the Maximum Likelihood statistical method and the numbers at internal node represent the percentage of 10,000 replicates in which the sequences grouped in the bootstrap test. The PvNRAMP protein sequences are in bold: PvNRAMP1 (Phvul.005G182000); PvNRAMP2 (Phvul.009G069700); PvNRAMP3 (Phvul.003G238600); PvNRAMP4 (Phvul.002G014300); PvNRAMP5 (Phvul.010G110500); and PvNRAMP7 (Phvul.009G127900). MtNRAMP1-MtNRAMP7 (*Medtr3g088460, Medtr3g088440, Medtr2g104990, Medtr3g102620, Medtr5g016270, Medtr8g028050*, and *Medtr4g095075*, respectively). The Phytozome code for *G.max* NRAMP genes were kept identifying the branches, except to *GmDMT1* (*Glyma17g18010*).
